# Antisense Oligonucleotides: An Emerging Area in Drug Discovery and Development

**DOI:** 10.3390/jcm9062004

**Published:** 2020-06-26

**Authors:** Karishma Dhuri, Clara Bechtold, Elias Quijano, Ha Pham, Anisha Gupta, Ajit Vikram, Raman Bahal

**Affiliations:** 1 Department of Pharmaceutical Science, University of Connecticut, Storrs, CT 06269, USA; karishma.dhuri@uconn.edu (K.D.); clara.bechtold@uconn.edu (C.B.); 2 Department of Genetics, Yale University, New Haven, CT 06520, USA; elias.quijano@yale.edu; 3 Department of Radiation Oncology, Vanderbilt University Medical Center, Nashville, TN 37232-5671, USA; hpham.cmu@gmail.com; 4 Department of Chemistry, Wesleyan University, Middletown, CT 06459, USA; agupta01@wesleyan.edu; 5 Division of Cardiovascular Medicine, Department of Internal Medicine, The University of Iowa, Iowa City, IA 52242, USA; ajit-vikram@uiowa.edu

**Keywords:** RNA, antisense oligonucleotides, chemical modifications, clinical trials

## Abstract

Antisense oligonucleotides (ASOs) bind sequence specifically to the target RNA and modulate protein expression through several different mechanisms. The ASO field is an emerging area of drug development that targets the disease source at the RNA level and offers a promising alternative to therapies targeting downstream processes. To translate ASO-based therapies into a clinical success, it is crucial to overcome the challenges associated with off-target side effects and insufficient biological activity. In this regard, several chemical modifications and diverse delivery strategies have been explored. In this review, we systematically discuss the chemical modifications, mechanism of action, and optimized delivery strategies of several different classes of ASOs. Further, we highlight the recent advances made in development of ASO-based drugs with a focus on drugs that are approved by the Food and Drug Administration (FDA) and the European Medicines Agency (EMA) for clinical applications. We also discuss various promising ASO-based drug candidates in the clinical trials, and the outstanding opportunity of emerging microRNA as a viable therapeutic target for future ASO-based therapies.

## 1. Introduction

Aberrant protein production or metabolism is associated with numerous devastating diseases and disorders [1,2,3,4]. As proteins are produced by decoding information stored in messenger RNA (mRNA), aberrant protein production can be regulated by targeting mRNA. Additionally, a greater understanding of RNA has unraveled its multifaceted roles. Until the advent of non-coding RNAs (ncRNAs), mRNA was only considered as the mediator between DNA and the ribosome for protein synthesis. Among ncRNAs, microRNA (miRNA) [5], transfer RNA-derived small RNA [6], pseudogenes [7], PIWI-interacting RNA [8], long ncRNAs (lncRNAs) [9], and circular RNAs [10] have been identified as critical regulators of biological functions through modulation of gene expression. Hence, the antisense strategy comprising of targeting pre-mRNA, mRNA, or ncRNAs can alter the production of disease-causing proteins for therapeutic interventions. Unlike small molecule-based protein targeting, antisense drugs exhibit their effect by Watson–Crick base pairing rules with target RNA sequence. This principle of Watson–Crick molecular recognition provides the antisense field more flexibility in RNA-based drug design and expedites its development, which is imperative for targeting a myriad of rare and genetic diseases [11]. The amalgamation of chemical structure modifications of oligonucleotides and diverse delivery platforms provides an additional boost to the antisense field. Recent United States Food and Drug Administration (FDA) approval of several nucleic acid-based drugs has further spurred interest in the antisense research. Presently, numerous antisense drug candidates are in clinical trials to treat cardiovascular, metabolic, endocrine, neurological, neuromuscular, inflammatory, and infectious diseases [12]. This review provides a brief overview of the structural modifications of new generation antisense oligonucleotides (ASOs), their mechanisms of action, delivery strategies, and comprehensive information about FDA-approved antisense therapies and current antisense-based drug candidates in clinical trials.

## 2. Oligonucleotide Modifications

In prior studies, ASOs based on phosphodiester backbone (also known as unmodified ASOs) were used to target RNA with moderate success. However, due to the presence of a phosphodiester bond, unmodified ASOs are susceptible to nuclease degradation [13]. In addition, the large size and charge of unmodified ASOs restrict their passive diffusion into the cell [14]. Hence, newer generation, chemically modified ASOs have been explored to increase their efficacy, enzymatic stability, and decrease immune response and off-target toxicity (Table 1).

### 2.1. Phosphorothioate (PS)

Phosphorothioate belongs to the first generation of ASOs that work by an mRNA cleavage-based mechanism [15]. In phosphorothioate (PS) ASOs, the non-bridging oxygen of the phosphate group is replaced by a sulfur group, resulting in the formation of a PS bond, which is resistant to nuclease-based degradation [16,17]. Compared to unmodified ASOs, the PS-ASOs strongly bind to serum proteins such as albumin, which further reduces their renal clearance and facilitates longer in vivo circulation [18]. Pharmacokinetic study in mice after intravenous (IV) administration of 30 mg kg^−1^ dose of PS-ASOs revealed 40% excretion in urine in 48 h [19]. Compared to unmodified ASOs, PS-ASOs show a predominant distribution in liver, kidney, and spleen when administered systemically, and demonstrate good cellular uptake. Following systemic administration in monkeys, PS-ASOs demonstrate biphasic plasma elimination with an initial half-life of 30–48 min, followed by a second-long half-life of 35–50 h [20]. However, repeated IV or intradermal administration of PS-ASOs in rodents elicits an immune reaction, highlighting the toxicity associated with them [21].

### 2.2. Phosphorodiamidate Morpholino Oligomer (PMO)

In phosphorodiamidate morpholino oligomers, the five-membered sugar moiety is substituted with a six-membered morpholine subunit, and each morpholine ring is inter-connected with phosphorodiamidate linkage [22]. PMOs are charge neutral and exert their antisense effect by steric hindrance or splice modulation. The absence of the carbonyl group in the PMO structure provides resistance against proteases and esterases [23]. The morpholine ring also increases its water solubility. Morpholinos have been extensively used in developmental biology for numerous antisense-based applications [24].

### 2.3. Peptide Nucleic Acids (PNA)

Peptide nucleic acids are synthetic nucleic acid mimics that contain neutral N-2-aminoethyl glycine units, with nucleobases connected by a flexible methyl carbonyl linker. Due to their neutral and unnatural backbone, PNAs are resistant to enzymatic degradation and possess a strong binding affinity with RNA sequences as compared to unmodified ASOs [25]. Significant challenges are associated with PNAs due to their low water solubility and decreased cellular uptake. Hence, various chemically modified PNAs, cationic PNAs [26], alpha and gamma guanidium PNAs [27], and lysine PNAs [28,29] have been developed to resolve these issues.

### 2.4. Locked Nucleic Acids (LNAs)

**Figure 1:**
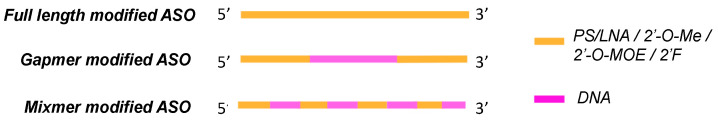
Antisense oligonucleotide (ASO) design. Chemically modified ASOs provide nuclease resistance and improved binding affinity to their target. Full length modified design represents chemical modifications throughout the sequence. Gapmer design includes a central region consist of DNA nucleotides and a stretch of LNA or 2′ modifications or PS nucleotides flanking both terminals of the sequence. Mixmer design contains LNA (or 2′ modifications) and DNA nucleotides present sequentially.

Locked nucleic acids contain a constrained methylene bridge between 2′ oxygen and 4′ carbon of the ribose ring and display a strong binding affinity to the target DNA or RNA sequences due to their preorganized structure. Each locked nucleic acid (LNA) modification increases the melting temperature of the duplex by 2–8°C [30]. LNAs express their antisense ability by steric hindrance mechanism. Diverse, comprehensive LNA designs, gapmer, mixmer, and other modifications have been evaluated for their antisense activity and compared with unmodified ASOs. Gapmers are LNA-DNA-LNA-based designs that contain continuous DNA nucleotides interspaced between two over hanged LNA nucleotides at the terminal regions (Figure 1). It has been well established that gapmer design stimulates RNase H1-based cleavage mechanism [31]. Mixmer designs contain interspaced DNA nucleotides between LNA nucleotides throughout the sequence and do not induce RNase H1 cleavage, but rather modulate mRNA expression through steric hindrance mechanism [32]. It has been noted that a minimum stretch of four LNA nucleotides at each terminal of the sequence (end block design) is adequate to provide a half-life of 15 h to LNAs, which is ~10-fold higher than that of unmodified ASOs [33]. 

#### Ribose Modifications

Chemical modification at the 2′ position of ribose sugars improves their binding affinity and provides resistance to enzymatic degradation. 2′ ribose modification includes 2′ fluoro (2′ F), 2′-O-methyl (2′-O-Me), and 2′-O-methoxyethyl (2′-O-MOE)-based ASOs [34]. Each change at the 2′ position increases the melting temperature of the duplex by 2 °C [35]. 2′-O-Me modification also decreases nonspecific protein binding during in vivo administration. As explained above, gapmer designs also apply to the 2′ ribose-modified class of ASOs. Here, in a gapmer design, a central region of unmodified antisense nucleotides is flanked by 2′ modified nucleotides at both ends. The unmodified nucleotides induce RNase H1 cleavage of the target RNA, and 2′ modified nucleotides improve the binding affinity of ASO to the region of interest and protect against endonucleases [36]. In a study by Shen et al. in mice, 2′ F-modified ASOs exhibits increase binding affinity to intracellular proteins like drosophila behavior/human splicing (DBHS) proteins, reducing DHBS proteins and resulting in hepatotoxicity [37]. Therefore, caution needs to be taken during in vivo studies as 2′ F modifications can lead to toxicity.

### 2.5. Nucleobase Modification

Nucleobase modifications are introduced to improve the properties of ASOs. Among nucleobase modifications, cytosine analogs have been used extensively. It has been noted that PS-ASOs that contain CpG dinucleotide stretches activate the toll-like receptor and cause immune stimulation [38]. Hence, 5-methyl cytosine-based analogs were used with PS-based chemistry to minimize the immune stimulation. Similarly, another cytosine analog, G-clamp has been used to increase the efficacy of ASOs. G-clamp modifications contain phenoxazine residues that form a total of five hydrogen bonds with complementary guanine nucleobase in the target sequence by Watson–Crick as well as Hoogsteen-base pairing [39]. It has been demonstrated that single G-clamp substitutions can increase the binding affinity by 23 °C [40,41].

## 3. Antisense Mechanism of Action

**Figure 2:**
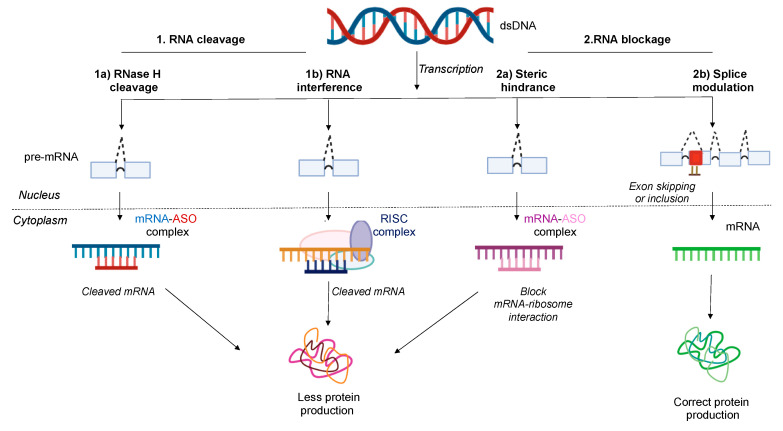
Mechanism of action of antisense oligonucleotides (ASOs): ASOs act by either causing (**1**) RNA cleavage or **(2)** RNA blockage. (**1a**) RNase H1 mediated cleavage, (**1b**) RNA interference (RNAi), (**2a**) Steric hindrance, and (**2b**) Splice modulation. (**1a**) ASO-mRNA heteroduplex recruits RNase H1 enzyme and this enzyme cleaves the target mRNA. (**1b**) mRNA degradation by siRNA associated with RNA inducing silencing complex (RISC). (**2a**) ASO-mRNA complex sterically blocks and prevents the interaction of mRNA with ribosomes for protein translation. (**2b**) is an example of splice switching oligonucleotides (SSO). Rectangles depict the coding exon regions separated by a curve depicting the non-coding intron region of the pre-mRNA. The red square represents the mutated region of the exon. The dashed line represents the splicing pattern of pre-mRNA. RNase H1 mediated cleavage, RNA interference, and steric hindrance mechanisms produce less protein, while splice modulation produce the correct form of protein. Phosphorothioate (PS) and 5′methylcytosine base modification induces mRNA cleavage. Peptide nucleic acids (PNA), 2′-O-methyl (2′-O-Me) and 2′-O-methoxyethyl (2′-O-MOE) modifications, phosphorodiamidate morpholino (PMO), locked nucleic acid (LNA) act on mRNA to sterically block its translation or these ASOs can act as SSO to modulate splicing pattern.

About four decades ago, Zamecnik and Stephenson first reported that 13-mer synthetic single-stranded ASOs could cause translational arrest by targeting Rous sarcoma virus mRNA [42]. After several studies, it has been well established that active ASOs are generally 15–20 nucleotides in length and can target complementary RNA by Watson–Crick base pairing without causing any significant off-target toxicity [43]. In addition, comprehensive studies established that the mechanism of synthetic ASOs can be of two types: (1) RNA cleavage and (2) RNA blockage (Figure 2) [44,45].

### 3.1. RNase H1 Mediated Degradation

ASOs target RNA, forming ASO-RNA heteroduplexes, which act as substrates for RNase enzymes present in the cytoplasm (Figure 2) [46]. RNases degrade RNA in the heteroduplex. Gapmer design contains a central region of unmodified nucleotides that aids in RNase H1 activity and flanking modified nucleotides increase its binding affinity and enzymatic resistance properties. Most of the drugs that have been FDA approved exert their antisense effect via RNases [47,48].

### 3.2. RNA Interference (RNAi)

Exogenous small interfering RNAs (siRNAs) are double-stranded 22 nucleotide RNA sequences with a 2-nucleotide overhang on the 3′ end of either strand [49]. This sequence associates with Argonaute 2 (Ago 2) enzyme to form the RNA induced silencing complex (RISC) where the passenger strand is degraded [50,51]. The remaining guide strand directs the RISC to the complementary mRNA region where Ago 2 enzyme cleaves the mRNA and exerts its gene silencing effect (Figure 2) [52]. RNAi works by a mechanism similar to the RNase H-dependent mechanism. The significant difference as compared to RNase-based degradation is that the siRNA is associated with the cleavage protein before interacting with the target site.

### 3.3. Steric Blockage

#### 3.3.1. Translation Arrest Due to Steric Hindrance

These classes of ASOs bind to target RNA sequence and cause translational arrest by inhibiting their interaction with the 40S ribosomal subunit or preventing their assembly on the 40S or 60S ribosomal subunit (Figure 2) [53,54]. ASOs based on steric hindrance do not activate RNase H1-mediated cleavage; therefore, the pre-mRNA structure is retained. Steric hindrance is directly related to the binding affinity of ASOs. An increase in binding affinity results in superior hybridization with target RNA, resulting in a translational arrest. In addition, synthetic single-stranded oligonucleotides of 20–25 nucleotides in length are designed to bind the miRNA and prevent their interaction with mRNA by steric hindrance-based mechanism, further controlling the gene expression [55]. A few studies have also reported that ASOs can bind to pre-miRNA in the nucleus and exert steric hindrance [56].

#### 3.3.2. Splice Modulation or Splice Switching-Based Mechanism

ASOs can also exert their effects by alternative splicing [57,58,59]. Splice modulation can be of two types: (1) exon skipping and (2) exon inclusions [60,61]. Frameshift mutations alter pre-mRNA splicing patterns that result in abnormal protein production or a translation arrest of full-length functional proteins [62,63]. In exon skipping, ASOs bind to the pre-mRNA transcripts, correct the disrupted reading frame and produce a short but functional protein [64]. Whereas in exon inclusion, ASOs bind to the pre-mRNA site and prevent the spliceosome and splicing factors from accessing the transcript sites (Figure 2) [65]. In 1993, for the first time, it was shown that 2′-O-Me precisely spliced the mutated beta-globin pre-mRNA in vitro to produce splice variant mRNA that restored hemoglobin production [66].

## 4. Delivery of ASOs.

While delivery of ASOs has always been a significant hurdle for their broad clinical applications, various strategies have been employed to deliver them [67,68,69].

### 4.1. Enhanced Stabilization Chemistry-Based Delivery

Alnylam extensively used enhanced stabilization chemistry (ESC) to deliver siRNA. In ESC, siRNAs are conjugated to N-Acetyl galactosamine (GalNAc) [70]. GalNac selectively targets the asialoglycoprotein receptor (ASGPR) that is highly expressed in hepatocytes [71]. GalNAc also stabilizes the siRNA conjugates in hepatocytes, plasma, as well as the lymphatic system, and reduces immune stimulation.

### 4.2. Nanoformulation-Based Delivery

Numerous polymeric nanoparticle-based delivery systems like PLGA, PBAE, and PEI have been used to deliver ASOs [72,73]. PBAE and PEI exert a ‘proton sponge’ effect that decreases the endosomal entrapment of ASOs and increases their delivery [74]. However, the advancement of these delivery systems to the clinic has been limited due to toxicity caused by excessive cationic charge as well as multiple non-specific interactions with serum and tissue proteins. On the contrary, PLGA being biocompatible and an FDA approved polymer, has been widely used to formulate nanoparticles and deliver ASOs [75,76].

### 4.3. Lipid-Based Delivery Systems

Several lipid-based delivery systems, lipoplexes, liposomes, and lipid nanoparticles (LNP) have been extensively used to deliver ASOs and siRNAs [77,78]. LNPs are typically coated with polyethylene glycol (PEG), which increases blood circulation time [79]. LNPs also show accumulation in the tumor microenvironment by the enhanced permeability and retention (EPR) effect. Patisiran contains LNP-based siRNA formulation. 

## 5. FDA-Approved Formulations

### 5.1. Fomivirsen (Vitravene^TM^)

In 1998, Fomivirsen was the first ASO-based drug developed by Ionis Pharmaceuticals. Fomivirsen received approval by the United States FDA for the treatment of retinitis caused by opportunistic cytomegalovirus (CMV) infection in immunocompromised AIDS patients (Table 2). CMV infection results in loss of vision in AIDS patients. Fomivirsen is a PS-based 21-mer ASO that hybridizes with the coding sequence of CMV mRNA from the major immediate-early region-2, which is responsible for CMV replication [80]. Fomivirsen inhibited CMV replication in a dose-dependent manner in preclinical studies. It exhibited a half-life of 78 h after intravitreal administration of 115 µg dose in monkeys [81]. Fomivirsen demonstrated clinical efficacy in a randomized clinical trial and delayed the progression of CMV infection in AIDS patients by 71 days as compared to 13 days in the deferred patient cohort [82]. However, the same period witnessed the success of the highly active antiretroviral therapy (HAART), which reduced the incidences of opportunistic inf-ections in AIDS patients and thus reduced market demand for Fomivirsen. Eventually, Fomivirsen was voluntarily withdrawn from the market by its manufacturers.

### 5.2. Mipomersen (Kynamro^TM^)

Homozygous familial hypercholesterolemia (HoFH) is an autosomal dominant genetic disorder that results in elevated low-density lipoprotein (LDL) levels. HoFH is caused by a mutation in the gene for LDL cholesterol receptor or pro-protein convertase subtilisin/kexin 9 (PCSK9) or apolipoprotein B (apo B) [83]. The elevated LDL levels result in high risk for developing coronary heart disease (CHD) or atherosclerosis at a young age. Mipomersen, an ASO developed by Genzyme, targets mRNA that encodes apo-B-100 mRNA and inhibits the synthesis of apolipoprotein B-100. It contains PS-based modifications throughout the sequence and five 2′-O-MOE-based nucleotides on both terminal ends. Mipomersen is administered subcutaneously at a dose of 200 mg once weekly and also used in combination with cholesterol-lowering drugs along with recommended lifestyle changes. In a placebo-controlled clinical trial study comprised of HoFH patients already on a lipid-lowering drug, Mipomersen reduced the LDL-cholesterol level by 25% compared to the placebo group. In addition, the patients treated with Mipomersen showed a reduction in serum VLDL cholesterol, non-HDL cholesterol, and lipoprotein(a) concentrations [84,85]. 

### 5.3. Nusinersen (Spinraza^®^)

Spinal muscular atrophy (SMA) is a genetic and autosomal recessive motor neuron disease caused by a homozygous deletion in the survival motor neuron 1 (SMN1) on chromosome 5q13 [86]. The SMN1 gene produces full-length mRNA that codes for functional SMN protein, which is required for normal functioning of motor neurons. Deficiency of SMN proteins results in the degeneration of lower muscle motor neurons, which leads to progressive paralysis due to muscle atrophy. In general, the SMN family contains two genes, SMN1 and SMN2. SMN2 differs from SMN1 by the presence of a thymine nucleotide instead of cytosine at the 840th position on the gene. This results in the exclusion of exon 7 from ~85% SMN2 pre-mRNA during splicing, resulting in the production of truncated SMN, which is unstable and rapidly degraded. However, the remaining 15% of SMN2 mRNA produces full-length functional SMN protein due to exon 7 inclusion [87]. Typically, everyone has the SMN1 and SMN2 gene; however, it becomes critical for SMA patients to present at least one copy of the SMN2 gene to allow for splice modulation to produce full-length SMN protein. Nusinersen is an ASO-based drug developed by Biogen for SMA treatment in pediatric and adult patients. It contains 2′-O-MOE and PS-based modifications [88]. Nusinersen hybridizes with SMN2 pre-mRNA and prevents the recruitment of splicing repressor, heterogeneous nuclear riboprotein A1 and A2, which favors the inclusion of exon 7 in the SMN2 mRNA and leads to increase in the production of functional SMN protein form [89,90]. Nusinersen treated SMA1 infants exhibited improved neuromuscular functions, possibly not requiring the use of permanent assisted ventilation as compared to the untreated SMA1 infants [91]. Clinically, a loading dose of 12 mg Nusinersen in a 5 mL volume is given intrathecally on the first, second, fourth, and ninth week, followed by maintenance therapy after every four-month interval [92]. One ongoing clinical trial is evaluating the efficacy of Nusinersen in improving the motor functions in adult SMN patients [93].

### 5.4. Patisiran (Onpattro^®^)

Transthyretin (TTR) is a tetrameric protein involved in the transport of thyroxin and retinol-binding protein vitamin A complex [94]. In healthy individuals, the TTR protein is present in cerebrospinal fluid and serum [95]. However, point mutations in the TTR gene produce aberrant TTR proteins that are more susceptible to misfolding and get deposited as TTR amyloid fibrils in extracellular spaces of the liver, heart, nerve, and gastrointestinal tract, eventually leading to organ dysfunction [96]. The aforementioned pathological condition is known as hereditary transthyretin-mediated amyloidosis (hATTR) and presents with severe symptoms of nausea, pain, and weakness. hATTR is estimated to affect around 50,000 patients worldwide. Patisiran is the first siRNA-based drug developed by Alnylam for hATTR treatment. Patisiran is a LNP-based formulation that is injected intravenously at a concentration of 2 mg/mL. In particular, D-Lin-MC3-DMA is an ionizable cationic lipid delivery vehicle used to encapsulate the siRNA. D-Lin-MC3-DMA has an acid dissociation constant (pKa) of 6.4 necessary to maintain a low surface charge to prevent early clearance of the siRNA from the body [97]. At a pKa of 6.4, the ionizable lipid group is positively charged, which aids in endosomal escape following endocytosis. Subsequently, siRNA is then released in the cell cytoplasm where it interacts with TTR mRNA, reducing TTR protein translation, thereby inhibiting the formation and deposition of amyloid plaques [98]. In a randomized, double-blinded placebo-controlled phase III study, patients treated with Patisiran showed an 80% reduction in serum TTR levels at doses between 0.15–0.5 mg kg^−1^ [99]. Three additional post-approval clinical trials are ongoing. The first trial is investigating the safety and efficacy of Patisiran in hATTR patients after liver transplant. The second study will assess the long-term safety of Patisiran treatment, and the third trial aims to evaluate and compare the efficacy of Vutrisiran to Patisiran for hATTR treatment [100,101,102].

### 5.5. Inotersen (Tegsedi^®^)

Inotersen was developed by Ionis Pharmaceuticals for hATTR treatment in 2018. Inotersen consists of gapmer design with five 2′-O-MOE nucleotides present at 5′ and 3′ ends with PS modifications present throughout the sequence. The 2′-O-MOE modification increases the ASO binding with the TTR mRNA without affecting its antisense activity. In a randomized, double-blinded placebo-controlled phase III clinical study, the safety and efficacy of subcutaneously administered Inotersen was evaluated in 112 patients and compared with 60 patients in a placebo group for 66 weeks. Composite scores based on the modified Neuropathy Impairment Score +7 (mNIS+7) and total score on the Norfolk Quality of Life-Diabetic Neuropathy (QOL-DN) questionnaire were the primary endpoints of the above-mentioned clinical study. In general, High mNIS+7 corresponds to inadequate response to therapy, and a high score on Norfolk QOL-DN is a measure of poor quality of life. The difference in the least-squares mean change from baseline to week 66 between the Inotersen and placebo-treated groups, which was −19.7 points for mNIS+7 and −11.7 points for the Norfolk QOL-DN, indicates the potential clinical efficacy of Inotersen. However, thrombocytopenia and glomerulonephritis were reported in a few patients during the study, warranting careful monitoring of hematological as well as a metabolic panel during the Inotersen-based treatment regimen [103].

### 5.6. Eteplirsen (Exondys 51^®^)

DMD is a fatal muscle degenerative disorder caused by mutations in the DMD gene, which encodes dystrophin. This partial gene deletion causes a reading frame shift resulting in an early stop codon that not only would prevent translation to functional dystrophin protein but, most importantly, induces mRNA destruction by nonsense-mediated RNA decay [104]. This results in loss of functional dystrophin, leading to muscle wasting and degeneration. Eteplirsen is a 30-mer oligonucleotide containing PMO-based chemical modifications developed by Sarepta Therapeutics for DMD treatment. Eteplirsen binds to exon 51 of DMD, which restores the reading frame of the dystrophin mRNA, translating short, but functional dystrophin [105,106]. In a preliminary study, two DMD patients were treated with Eteplirsen, and five patients from the placebo group were given saline as control. The patients receiving Eteplirsen showed an increase in dystrophin levels as quantified by immunohistochemistry-based endpoint analysis [107]. The clinical efficacy of Eteplirsen was evaluated in a small cohort of patients, wherein a marginal elevation from 0.16% at baseline to 0.48% at 48 weeks in dystrophin protein levels was observed [108]. Eteplirsen received FDA approval after considering the lack of available treatment options, severity, and progressive nature of the DMD disease. Additional post-approval clinical trial studies to validate the efficacy of Eteplirsen in decelerating the progression of DMD disease are ongoing as mandated by the FDA [109].

### 5.7. Golodirsen (Vyondys 53^TM^)

Golodirsen is developed by Sarepta Therapeutics for the treatment of DMD. Golodirsen utilizes a similar therapeutic strategy (PMO chemistry) as the one described for Eteplirsen. Golodirsen however, addresses patients with a different deletion in the dystrophin gene. It hybridizes with DMD pre-mRNA and cause exon 53 skipping leading to the production of short but functional dystrophin protein required for muscle activity. The safety and efficacy of Golodirsen was evaluated in a randomized, double-blind, placebo-controlled study in two phases. In the first phase, all 25 patients treated with increasing doses of Golodirsen for 48 weeks showed a 16-fold increase in dystrophin protein levels over baseline as compared to the placebo group. Golodirsen was found to be well-tolerated in patients after weekly IV infusion for 48 weeks. Muscle biopsies also indicated an increase in dystrophin production in the Golodirsen treated group as compared to the placebo group [110].

### 5.8. Givosiran (Givlaari^®^)

Acute hepatic porphyria (AHP) is a genetic disorder in which the enzyme delta-aminolevulinate synthase 1 (ALAS1) is produced in excess. The excess of ALAS1 enzyme accumulates neurotoxins, aminolevulinic acid (ALA), and porphobilinogen (PBG), which results in intense abdominal pain, nausea, and seizures [111]. Givosiran, developed by Alnylam Pharmaceuticals, is the second FDA approved siRNA drug for the treatment of AHP (Table 2). This siRNA is conjugated to GalNAc to achieve liver-specific delivery, where it leads to decreased ALAS1 enzyme, eventually reducing the levels of ALA and PBG [112]. A clinical study of Givosiran consisting of a total of 94 patients, showed that 74% of the patients receiving Givosiran experienced fewer incidences of porphyria. Givosiran is given by a subcutaneous route once a month. Common side effects include nausea and reactions at the injection site. Liver and kidney functions and allergic reactions are required to be monitored during and after treatment. The pharmacokinetics and long-term safety of Givosiran in acute intermittent porphyria treatment (AIP) is under clinical investigation [113].

### 5.9. Milasen

Milasen is a personalized ASO that was specifically designed to treat a six-year-old child suffering from neuronal ceroid lipofuscinosis 7 (CLN7) that affects the central nervous system (Table 2). This rare and fatal neurodegenerative condition, also known as Batten disease, leads to loss of vision, dysarthria, and dysphagia. Whole-genome sequencing of the patient identified the insertion of SVA (SINE-VNTR-Alu) retrotransposon in the MFSD8 gene (also called CLN7) that alters the splicing of transcripts. A series of ASOs were tested in patient fibroblasts to target cryptic splice sites in the MFSD8 pre-mRNA and restore normal (exon 6–7 ) splicing. The lead ASO, Milasen, is 22-nucleotides long and was developed using the same chemical modifications as present in Nusinersen. Milasen did not show any toxicity during pre-clinical studies in rats. Based on promising preliminary pre-clinical results, Milasen was granted an expedited N-of-1 approval for use in this patient. Excitingly, Milasen reduced the frequency of the seizures and effectively improved the quality of life of the patient [114]. Development of Milasen for targeting Batten disease in an N-of-1 patient is a promising example of RNA-based personalized medicine.

## 6. Potential Drug Candidates in Clinical Trials

The list of the antisense formulations in clinical trials is expanding with many candidates successfully reaching phase III clinical trials (Table 3). Here, we discuss a few selected investigational drugs that showed promise in phase III clinical trials.

### 6.1. Tominersen

Huntington disease (HD) is an autosomal dominant, progressive neurodegenerative disorder characterized by dystonia, cognitive dysfunction, and behavioral difficulties [115]. In HD, the CAG trinucleotide repeat expansion results in mutant Huntington (mHTT) protein with extended polyglutamine tract [116]. mHTT accumulates in neurons, thereby affecting their normal function [117]. Tominersen (RG6042) is an ASO developed by Ionis Pharmaceuticals for targeting mHTT mRNA to prevent protein production. The disease-modifying potential of Tominersen was evident in phase I and II clinical trials evaluated in 46 patients with early-stage HD. Patients received a monthly dose of Tominersen (10, 30, 60, 90, 120 mg) or a placebo, administered intrathecally for three months. An average 40% reduction in mHTT proteins was observed in the cerebrospinal fluid at a dose of 90 and 120 mg. The drug candidate showed good tolerability in patients and showed no adverse effects at high doses.

### 6.2. Tofersen

Superoxide dismutase (SOD1) enzyme plays an essential role in scavenging free radicals generated in the body. Mutation in the SOD1 gene results in the production of dysfunctional SOD1 that accumulates as toxic protein in the cells and results in familial amyotrophic lateral sclerosis (ALS). Tofersen is an ASO designed to target mutated SOD1 mRNA to prevent protein production, thereby slowing ALS progression. A phase I and II double-blind, randomized, placebo-controlled trial showed a reduction in dysfunctional cerebrospinal fluid SOD1 concentrations in the Tofersen treated group [118]. Tofersen treatment also results in an improvement in respiratory and muscle function as compared to placebo control. Tofersen is developed by Biogen and a phase III trial is ongoing to examine the clinical efficacy of Tofersen in SOD1-ALS patients.

### 6.3. Volanesorsen (Waylivra^®^)

Familial chylomicronemia syndrome (FCS) is caused by mutations in the lipoprotein lipase (LPL) gene. This condition impairs the usual break down of fats resulting in increased accumulation of fats in the blood. Abdominal pain, eruptive xanthoma, pancreatitis, hepatosplenomegaly, and lipemia retinalis are serious manifestations of FCS. Volanesorsen, developed by Ionis Pharmaceuticals, inhibits hepatic APOC3 mRNA, resulting in reduced plasma triglyceride and apolipoprotein C-III levels. In a 53-week placebo-controlled clinical trial, Volanesorsen was given once weekly subcutaneously to 66 patients with FCS. The total triglyceride levels reduced by 76.5%, and the apolipoprotein C-III levels reduced by 84.2% from baseline at three months in patients treated with Volanesorsen [119]. During the trial, two patients showed a low platelet count of 25,000 platelets per microliter of blood (normal platelet count is 140,000–450,000 platelets per microliter of blood). The platelet count returned to normal levels in the two patients 33 days after discontinuing the drug. Mild to moderate injection site reactions were observed in 20 patients receiving Volanesorsen. Due to significant lipid-lowering potential, Volanesorsen received conditional marketing authorization in Europe to treat patients with genetic FCS and patients at high risk of acute pancreatitis in whom lipid-lowering therapy has not been effective.

### 6.4. Alicaforsen

Pouchitis is inflammation caused in the lining of a pouch created during complications in surgery or ulcerative colitis. Intercellular adhesion molecule-1 (ICAM-1), is a cell surface receptor that directs the white blood cells in circulation to sites of inflammation and increases the inflammatory response in pouchitis. Alicaforsen is an ASO developed by Atlantic Healthcare that hybridizes with ICAM-1 mRNA and reduces its levels. In clinical trials, treatment with Alicaforsen reduces inflammation and stool frequency, thereby improving the quality of life. Endoscopy showed an improvement in the underlying tissue as well [120].

### 6.5. Vutrisiran

Vutrisiran is an investigational drug developed by Alnylam Pharmaceuticals for the treatment of hATTR [121]. The mechanism of action of this drug is the same as Patisiran. Vutrisiran is administered by the subcutaneous route (25 mg every 3 months for 2 years) and phase III clinical studies are ongoing to determine its therapeutic effect.

### 6.6. Fitusiran

Hemophilia is an X-linked bleeding disorder caused due to deficiency of blood clotting factor VIII or factor IX [122]. Fitusiran, an siRNA-based investigational drug developed by Alnylam Pharmaceuticals, acts by inhibiting antithrombotic (AT) mRNA in the liver. In phase II clinical trials, 25 Hemophilia A and B patients were given Fitusiran (50 mg) once a month. Interestingly, 81% knockdown of AT mRNA corresponded to 49–100% reduction in bleeding frequency. The annualized bleeding rate (ABR) of patients improved from 12 to 1.7 after treatment with Fitusiran for 13 months [123].

### 6.7. Inclisiran

Patients with heterozygous familial hypercholesterolemia (HeFH) have high serum LDL levels, which predisposes them to atherosclerotic cardiovascular disease [124]. Though monoclonal antibodies targeting PCSK9 reduce LDL levels by 50%, these antibodies require weekly administration and only target circulating PCSK9. Inclisiran is a siRNA drug developed by The Medicines Company for targeting PCSK9 mRNA for the treatment of HeFH in patients. In a phase III clinical trial, Inclisiran was administered to 242 patients, compared to 240 patients receiving placebo subcutaneously on days 1, 90, 270, and 450. Patients receiving Inclisiran showed a 39.7% decrease in LDL, while the placebo group showed an increase of 8.2% after 16 months of treatment [125]. The LDL levels were below 100 mg per deciliter in about 65% of the patients treated with Inclisiran. Inclisiran only requires two doses a year, which is an advantage over weekly monoclonal antibody therapy. Following the promising results of Inclisiran in phase III clinical trials, Novartis acquired The Medicines Company and Inclisiran rights in January 2020.

## 7. ASOs Targeting microRNA

**Figure 3:**
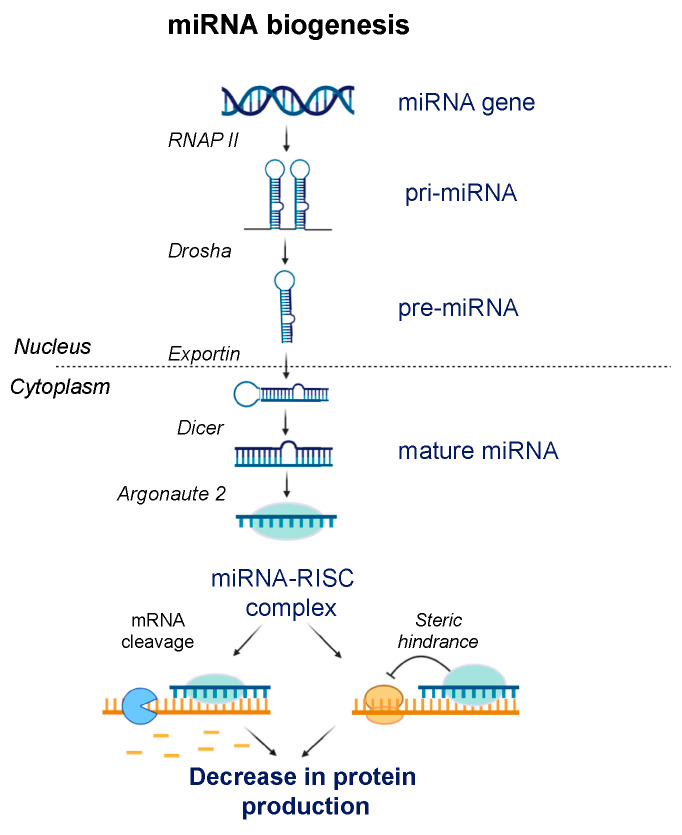
miRNA biogenesis and mechanism of action. miRNA is transcribed by RNA polymerase II (RNAP II) to form double stranded hairpin loop structure called pri-miRNA, which gets cleaved by nuclease Drosha to form pre-miRNA. Exportin transports the pre-miRNA to the cytoplasm where it is further processed by Dicer to form a single stranded mature miRNA. The mature miRNA is uploaded in the RNA induced silencing complex (RISC) where it associates with Argonaute 2 protein. This miRNA-RISC complex interacts with the seed region of the mRNA and regulates the mRNA translation by either mRNA cleavage or by steric hindrance.

miRNAs are 20–25 nucleotides long ncRNAs that play critical roles in the development and establishment of cell identity, and atypical expression of miRNAs leads to various malignant and non-malignant disorders. Miravirsen was developed by Roche for targeting miR-122 for hepatitis C virus (HCV) infection [165]. Miravirsen contains LNA as well as PS-based chemistry. Severe side effects, however, halted clinical trials of Miravirsen. Similarly, Regulus Therapeutics developed antimiR-21 (RG-012) for Alport syndrome to decrease the rate of progression of renal fibrosis [166]. RG-012 received orphan drug status in the US and Europe. MicroRNAs that promote carcinogenesis and metastasis have also been targeted by the ASOs [167]. One important example is miR-155, which has been shown to be up-regulated in many subtypes of lymphoma, including diffuse large B-cell lymphoma (DLBCL) [168,169]. Cobomarsen (MRG-106) is an LNA-based miR-155 inhibitor developed by miRagen Therapeutics and is currently in phase II trials to treat cutaneous T-cell lymphoma as well as adult T-cell lymphoma and leukemia [170]. The biogenesis and mechanism of action of miRNA are illustrated below (Figure 3).

## 8. Conclusions

The excitement surrounding antisense oligonucleotide-based drug discovery and development had previously waned due to lack of clinical efficacy even at high ASO doses [171]. Incredible research efforts, however, were made at the interface of chemistry and biology to improve the biophysical and biological attributes of the ASOs [172]. This advanced their in vivo efficacy with minimal off-target effects, leading to several FDA and EMA approvals of ASO-based drugs. The revelation of new molecular targets has also invigorated medical and biological sciences to exploit different classes of ASOs to treat rare and genetic disorders which were previously deemed untreatable by conventional small-molecule-based therapies. In addition, different classes of ASOs are being tested successfully to target the same disease (Patisiran and Inotersen are used to treat hATTR, and Golodirsen and Eteplirsen for treating DMD) to broaden their clinical application based on patient history and clinical outcome.

Overall, ASO-based investigational drugs possess numerous benefits; flexibility in design due to Watson–Crick base recognition, optimized synthesis protocols as well as broad quality control during the development phase. Since ASO-based drugs target the root cause of specific diseases, they have to be administered weekly (Mipomersen) or once every four months (Nusinersen) to establish their efficacy as compared to small molecule-based drugs, which have to be administered daily in most cases. Excitingly, this field has gathered momentum in the last several years as ASO-based drugs received FDA and EMA approval for clinical applications. In addition, the rapid translation of Milasen from proof-of-concept to clinical use within months is an exceptional and enthusiastic example of the development of an ASO-based precision medicine that can improve a patient’s quality of life by reducing disease severity. Overall, ASOs can be employed as personalized medicine (Milasen), as well as for the treatment of a sizeable patient population (Inclisiran).

However, several challenges need to be overcome to broaden the clinical use of ASOs, principal among them being their delivery. ASOs typically accumulate in organs like kidney, liver, and spleen, resulting in toxicity. Since several delivery platforms have been developed, a more concerted effort needs to be employed to explore delivery strategies that can deliver ASOs in maximum effective dose to target organs without off-target accumulation. Optimized delivery platforms will help reduce ASO dose, increase efficiency, and allow tissue-specific targeting that can further minimize their toxicity. In considering the delivery platforms, it is also critical to be mindful of scalability and reproducibility at the industrial scale. Similar caution must be used when designing ASOs given their potential for self-hybridization and off-target binding.

In addition to rare and genetic diseases, several attempts have been made in the past to develop novel antiviral-based ASO drugs [173]. The world is witnessing a devastating pandemic of coronavirus disease 2019 (COVID-19) caused by a novel coronavirus, SARS-CoV-2. Several ASO-based drugs are under evaluation by RNA-based companies like Alnylam Pharmaceuticals to target SARS-CoV-2. Since the viral genome is known, multiple ASOs can be designed and tested to target different viral regions; spike (S) protein, membrane (M) protein, envelop (E) protein, and nucleocapsid (N) for impairing viral function [174]. In addition, ASO therapies are in process to provide symptomatic relief by suppressing cytokines. A few studies are centered on the development of novel ASOs to target toll-like receptor (TLR) and trigger the production of protective interferons to combat the virus [175]. In summary, ASOs have seen tremendous progress in recent years and gained stature in the pharmaceutical market parallel to small molecule-based drugs. Hence, it is not too early to envision that in the future, in conjunction with novel delivery platforms, ASO-based drugs will be used for treating widespread diseases and disorders with maximum efficacy and minimal toxicity.

## Figures and Tables

**Table 1:** Chemical modifications of antisense oligonucleotides (ASO).

Name	Structure	Mechanism	Properties
**Phosphate modification**			
Phosphorothioate (PS)	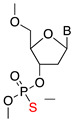	RNase H1 cleavage	Enzymatic stability
**Sugar phosphate** **modification**			
Phosphorodiamidate morpholino (PMO)	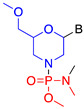	Steric hindrance/splice modulation	Improved aqueous solubility, higher binding affinity
Peptide nucleic acid (PNA)	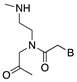	Steric hindrance/splice modulation	Enzymatic stability, higher binding affinity, no immune activation
**Sugar modification**			
Locked nucleic acid (LNA)	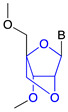	Steric hindrance/RNase H1 cleavage	Higher binding affinity, enzymatic stability
2′-O-methyl (2′-O-Me)	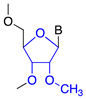	Steric hindrance/splice modulation	Higher binding affinity, enzymatic stability, reduced immune stimulation
2′-O-methoxyethyl (2′-O-MOE)	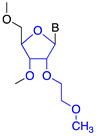	Steric hindrance/splice modulation	Higher binding affinity, enzymatic stability, reduced immune stimulation
2′fluoro (2′ F)	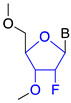	Steric hindrance/splice modulation	Higher binding affinity
**NucleoBase modification**			
5′methylcytosine	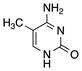	RNase H1 cleavage	Higher binding affinity, no immune stimulation
G-clamp	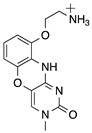	Steric hindrance	Higher binding affinity

**Table 2:** FDA approved drugs.

Drug	Chemistry	Route	Target	Indication	Approval	Designation	Company
Fomivirsen (Vitravene^TM^)	PS	IVT	CMV mRNA	CMV infection	FDA (1998)	-	Ionis
Mipomersen (Kynamro^TM^)	2′-O-MOE, PS, 5-methyl cytosine	SC	apo-B-100 mRNA	HoFH	FDA (2013)	Orphan	Genzyme
Nusinersen (Spinraza^®^)	2′-O-MOE, PS, 5-methyl cytosine	ITH	SMN2 pre-mRNA	SMA	FDA (2016), EMA (2017)	Orphan	Biogen
Patisiran (Onpattro^®^)	siRNA	IV	TTR mRNA	hATTR	FDA (2018), EMA (2018)	Orphan	Alnylam
Inotersen (Tegsedi^®^)	2′-O-MOE, PS	SC	TTR mRNA	hATTR	FDA (2018), EMA (2018)	Orphan	Ionis
Eteplirsen (Exondys 51^®^)	PMO	IV	exon 51	DMD	FDA (2016), EMA (2018)	Orphan	Sarepta
Golodirsen (Vyondys 53^TM^)	PMO	IV	DMD pre-mRNA	DMD	FDA (2019)	Orphan	Sarepta
Givosiran (Givlaari^®^)	siRNA	SC	ALS1 mRNA	AHP	FDA (2019), EMA (2020)	Orphan	Alnylam
Milasen	2′-O-MOE, PS, 5-methyl cytosine	ITH	intron 6 spice acceptor cryptic site	CLN7	FDA * (2018)	Orphan	Boston Children’s Hospital

* Milasen is a personalized medicine developed for a single patient. IV—Intravenous, SC—Subcutaneous, IVT—intravitreal, ITH—Intrathecal.

**Table 3:** Potential drug candidates in clinical trials.

Drug Candidate NCT ID	Chemistry/Delivery	Target	MOA	Route	Company	Indication	Ref.
Phase I status							
ARRx 03300505	cEt gapmer	Androgen receptor mRNA	RNase H1	IV	Rogel Cancer Center	Prostate cancer	[126]
RG-012 03373786	-	miR-21	antimiR	SC	Genzyme	Alport syndrome	[127]
QR-010 02564354	-	CFTR mRNA	Splice modulation	IN	ProQR	Cystic fibrosis	[128]
ISTH0036 02406833	LNA	TGF beta 2	RNase H1	IVT	Isarna	Primary open angle glaucoma	[129]
ARO-APOC3 03783377	siRNA-GalNAc	ApoC-III mRNA	RNAi	SC	Arrow head	HTG, FCS	[130]
ARO-ANG3 03747224	siRNA-GalNAc	Angiopoietin-like protein 3 mRNA	RNAi	SC	Arrow head	Dyslipidemias, FH, HTG	[131]
QPI-1007 01064505	siRNA	Caspase 2 mRNA	RNAi	IVT	Quark	Anterior ischemic optic neuropathy, glaucoma	[132]
ALN-AAT02 03767829	siRNA-GalNAc	Alpha-1 antitrypsin mRNA	RNAi	SC	Alnylam	Alpha-1 antitrypsin deficiency liver disease	[133]
MEDI1191 03946800	mRNA LNP	IL-12	coding mRNA	IT	Med Immune	Solid tumors	[134]
Phase II status							
ISIS-FGFR4RX 02476019	2′-O-MOE-PS	FGFR4 mRNA	RNase H1	SC	Ionis	Obesity	[135]
IONIS DGAT2Rx 03334214	2′-O-MOE-PS	DGAT 2 mRNA	RNase H1	SC	Ionis	Hepatic steatosis	[136]
IONIS-PKK Rx 04307381	2′-O-MOE-PS	Pre kallikrein mRNA	RNase H1	SC	Ionis	Hereditary angioedema	[137]
ISIS-GCGRRx 02583919	2′-O-MOE GalNAc	Glucagon receptor mRNA	RNase H1	SC	Ionis	Type 2 diabetes	[138]
Custirsen 00327340	siRNA	Clusterin mRNA	RNase H1	IV	Achieve Life Sciences	Prostate cancer	[139]
OGX-427 01454089	LNA	Hsp27 mRNA	RNase H1	IV	Achieve Life Sciences	Metastatic bladder cancer, urinary tract neoplasms	[140]
ISIS681257 03070782	2′-O-MOE-PS	Lp(a) mRNA	RNase H1	SC	Akcea	Elevated lipoprotein (a), cardiovascular disease	[141]
AKCEA-ANGPTL3-LRx 03514420	cEt gapmer	ANGPTL3 mRNA	RNase H1	SC	Akcea	Familial partial lipodystrophy	[142]
ISIS678354 03385239	GalNAc-ASO	ApoC-III mRNA	mRNA inhibitor	SC	Akcea	HTG, cardiovascular diseases	[143]
Danvatirsen 02983578	2′-O-MOE-PS	STAT3 mRNA	RNase H1	IV	M.D. Anderson Cancer Center	Refractory pancreatic, NSCLC, colorectal cancer	[144]
Cobomarsen (MRG106) 03713320	LNA	miR-155	antimiR	IT	miRagen	Cutaneous T-cell lymphoma	[145]
Remlarsen 03601052	2′-O-MOE	miR-29	miRNA mimic	ID	miRagen	Keloid	[146]
Cemdisiran 03841448	siRNA-GalNAc	C5 mRNA	RNAi	SC	Alnylam	IgA nephropathy glomerulo nephritis	[147]
ARO-AAT 03946449	siRNA-GalNAc	Alpha-1 antitrypsin mRNA	RNAi	SC	Arrow head	Alpha 1-antitrypsin deficiency	[148]
PF-655 01445899	siRNA	RTP801	RNAi	IVT	Quark	Diabetic macular edema	[149]
AZD8601 03370887	mRNA LNP	VEGF-A mRNA	coding mRNA	EI	Astra Zeneca	Heart failure	[150]
DS-5141b 02667483	ENA	Dystrophin mRNA exon 45	Splice modulation	SC	Daiichi Sankyo	DMD	[151]
SB010 01743768	-	GATA-3	DNAzyme	I	Sterna Bio.	Asthma	[152]
Miravirsen 01727934	LNA	miR-122	antimiR	SC	Santaris	Hepatitis C	[153]
BP1001 02923986	LNA	Grb2	-	IV	Bio-Path Holdings	Leukemia	[154]
Phase III status							
Tominersen 03842969	2′-O-MOE-PS	HTT mRNA	RNase H1	ITH	Ionis	Huntington’s disease	[155]
Tofersen 02623699	2′-O-MOE-PS	SOD1 mRNA	RNase H1	ITH	Ionis	Amyotrophic lateral sclerosis	[156]
IONIS-TTR RX 02175004	2′-O-MOE-PS	TTR mRNA	RNase H1	SC	Ionis	Familial amyloid poly neuropathy	[157]
Volanesorsen 02658175	2′-O-MOE-PS	ApoC-III mRNA	RNase H1	SC	Ionis	FCS hyperlipo proteinemia type 1	[158]
AKCEA-TTR-LRx 04136171	siRNA GalNAc	TTR mRNA	RNase H1	SC	Ionis	ATTR cardio myopathy	[159]
Alicaforsen 02525523	PS	ICAM-1 mRNA	RNase H1	E	Atlantic	Pouchitis	[160]
Vutrisiran 04153149	siRNA-GalNAc	TTR mRNA	RNAi	SC	Alnylam	ATTR with cardio myopathy	[161]
Fitusiran 03549871	siRNA-GalNAc	Anti-thrombin mRNA	RNAi	SC	Genzyme	Hemophilia	[162]
QPI-1002 03510897	siRNA	p53 mRNA	RNAi	IV	Quark	Cardiac surgery	[163]
Inclisiran 03814187	siRNA-GalNAc	PCSK9 mRNA	RNAi	SC	The Medicines Company	Heterozygous FH	[164]

MOA—mechanism of action, IV—Intravenous, SC—Subcutaneous, IN—Intranasal, IVT—intravitreal, IT—Intratumoral, ID—Intradermal, EI—Epicardial, ITH—Intrathecal, I—Inhalation, E—Enema, cEt—Constrained ethyl, CFTR—Cystic fibrosis transmembrane conductance regulator, TGF—Transforming growth factor, Apo—Apolipoprotein, HTG—Hypertriglyceridemia, FCS—Familial chylomicronemia syndrome, IL—Interleukin, FGFR4—Fibroblast growth factor receptor 4, DGAT—Diacylglycerol transferase, Hsp—Heat shock protein, Lp(a)—Lipoprotein (a), ANGPTL3—Angiopoietin-like protein 3, STAT3—Signal transducer and activator of transcription 3, NSCLC—Non-small cell lung cancer, C5—Complement C5, VEGF—Vascular endothelial growth factor, ENA—Ethylene-bridged nucleic acid, DMD—Duchenne muscular dystrophy, HTT—Huntingtin, ICAM—Intercellular adhesion molecule, ATTR—Transthyretin amyloidosis, p53—Tumor protein, FH—Familial hypercholesterolemia.
